# Creation of a Merkel cell polyomavirus small T antigen-expressing murine tumor model and a DNA vaccine targeting small T antigen

**DOI:** 10.1186/2045-3701-3-29

**Published:** 2013-07-15

**Authors:** Bianca Gomez, Liangmei He, Ya Chea Tsai, T-C Wu, Raphael P Viscidi, Chien-Fu Hung

**Affiliations:** 1 Departments of Pathology, Johns Hopkins Medical Institutions, Baltimore, MD, USA; 2 Pediatrics, Johns Hopkins Medical Institutions, Baltimore, MD, USA; 3 Obstetrics and Gynecology, Johns Hopkins Medical Institutions, Baltimore, MD, USA; 4 Molecular Microbiology and Immunology, Johns Hopkins Medical Institutions, Baltimore, MD, USA; 5 Oncology, Johns Hopkins Medical Institutions, Baltimore, MD, USA; 6 Departments of Pathology and Oncology, The Johns Hopkins University School of Medicine, CRB II Room 307, 1550 Orleans Street, Baltimore, MD 21231, USA

**Keywords:** DNA vaccine, Gene therapy, Merkel cell polyomavirus, Small T antigen, Merkel cell carcinoma

## Abstract

**Background:**

Merkel cell polyomavirus (MCPyV) is a DNA virus expressing transcripts similar to the large T (LT) and small T (ST) transcripts of SV40, which has been implicated in the pathogenesis of Merkel cell carcinoma (MCC), a rare and highly aggressive neuroendocrine skin cancer. MCPyV LT antigen expression was found to be a requirement for MCC tumor maintenance and ST protein also likely contributes to the carcinogenesis of MCC. Previously, we have identified the probable immunodominant epitope of MCPyV LT and developed a DNA vaccine encoding this epitope linked to calreticulin. The LT-targeting DNA vaccine generated prolonged survival, decreased tumor size and increased LT-specific CD8+ T cells in tumor-bearing mice.

**Results:**

In this study, we developed a MCPyV ST-expressing tumor cell line from B16 mouse melanoma cells. We then utilized this ST-expressing tumor cell line to test the efficacy of a DNA vaccine encoding ST. In ST-expressing tumor-bearing mice, this vaccine, pcDNA3-MCC/ST, generated a significant number of ST antigenic peptide-specific CD8+ T cells and experienced markedly enhanced survival compared to mice vaccinated with empty vector.

**Conclusions:**

The formation of an effective vaccine against MCPyV has the potential to advance the field of MCC therapy and may contribute to the control of this severe malignancy through immunotherapy. Both of the innovative technologies presented here provide opportunities to develop and test MCPyV-targeted therapies for the control of Merkel cell carcinoma.

## Background

Primarily occurring among elderly and immunosuppressed individuals, Merkel cell carcinoma is a rare, although highly aggressive neuroendocrine skin cancer. Typically suggestive of a viral etiology, this pattern of occurrence led to the discovery of Merkel cell polyomavirus (MCPyV). MCPyV, a DNA virus expressing transcripts similar to the large T (LT) and small T (ST) transcripts of SV40, has been implicated in the pathogenesis of Merkel cell carcinoma [1]. The pivotal study by Feng et al detected MCPyV in 80% of MCC tumors compared to 16% of control skin tissues [1] and other research groups have corroborated this finding [2,3].

MCPyV DNA was found to be integrated into the tumor genome of MCCs in a monoclonal pattern indicating that MCPyV is the etiologic factor in the majority of MCCs as viral integration likely occurs prior to clonal expansion [1]. The further investigation of MCPyV T antigen sequences in MCCs led to key insights into the potential mechanisms of viral-mediated oncogenesis. Remarkably, the manner in which MCPyV DNA integrates in the host genome renders the virus incapable of replicating. Additionally, although the integrated LT protein is prematurely truncated, it retains the ability to bind the tumor suppressor protein, retinoblastoma (Rb) [4]. These characteristic MCC tumor-specific mutations of MCPyV are indicative of a causal role for MCPyV in the pathogenesis of MCC.

Providing additional evidence of MCPyV as the infectious cause of MCC, MCPyV LT antigen expression was found to be required for MCC tumor maintenance [5]. The dependence of MCC on tumor antigen expression makes LT antigen an ideal immunotherapeutic target for the treatment of MCC. In addition to LT, the MCPyV ST protein is also likely to contribute to the carcinogenesis of MCC. ST shares the same amino terminus as LT, but has a distinct carboxy terminus containing a protein phosphatase 2A binding site, which has been shown to be important in other polyomaviruses for virus-induced transformation [6]. ST is expressed in most MCC tumors and is required for tumor cell growth [7]. ST acts as an oncoprotein by targeting the 4E-BP1, a translational regulator whose inhibition is required for MCPyV transformation.

In previous studies, we identified the probable immunodominant epitope of MCPyV LT as the span of amino acids 19-27 [8]. Furthermore, we demonstrated that tumor-challenged mice injected with a DNA vaccine encoding the 19-27 peptide linked to calreticulin (CRT) experienced prolonged survival and decreased tumor size [8]. The DNA vaccine also generated the highest number of LT-specific CD8+ T cells in mice. In the present study, we intended to utilize a DNA vaccine encoding the ST antigen to generate an antitumor effect in a murine tumor model expressing ST. Similar to our previous study targeting LT, we found that vaccination with ST DNA resulted in prolonged survival in *in vivo* protection and treatment experiments in ST-expressing tumor-challenged mice. Our data have significant implications for future clinical translation.

## Results

### Creation of a murine tumor model that expresses MCPyV ST antigen, B16MCC/ST

**Figure 1:**
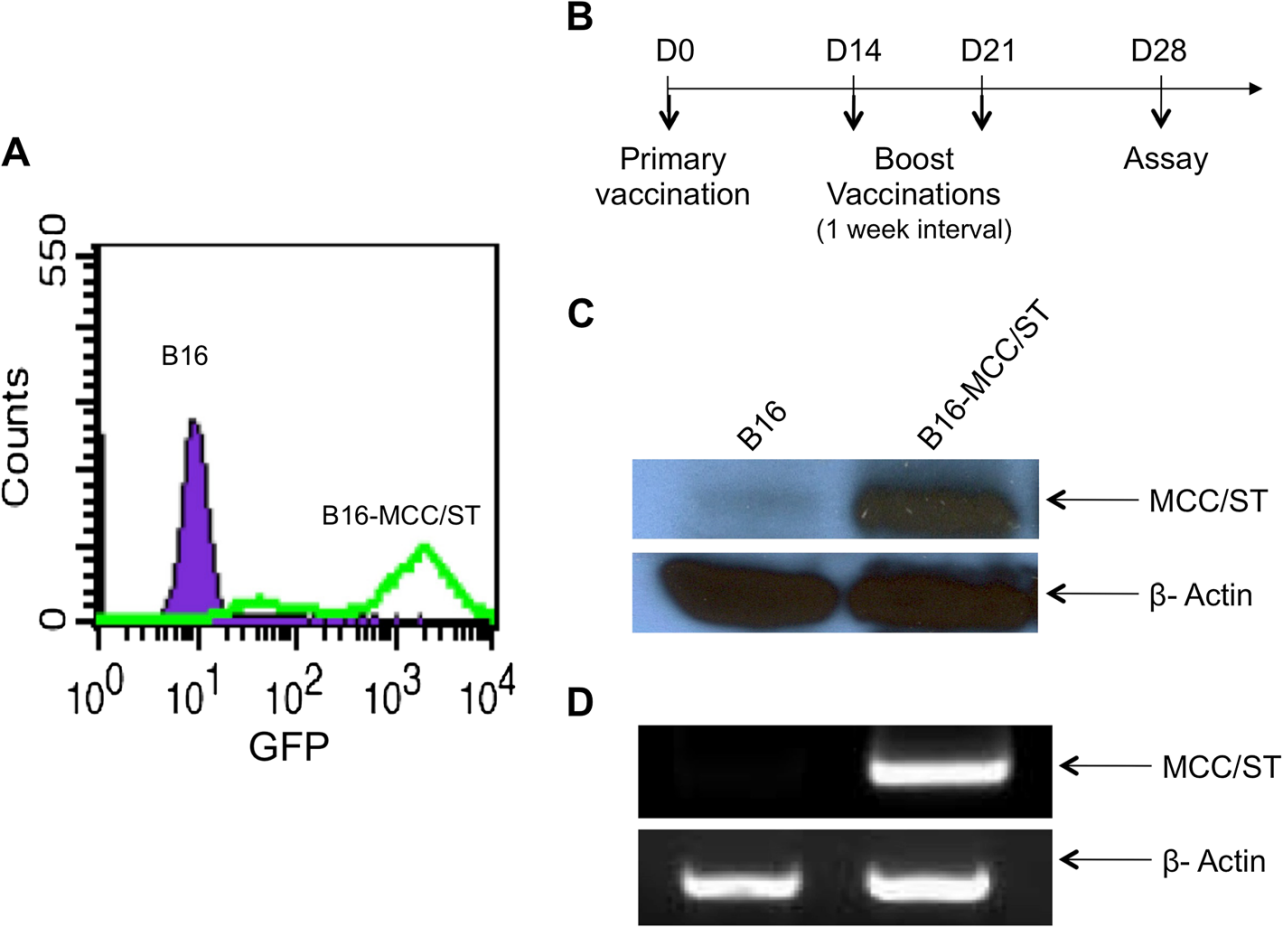
**Generation and characterization of ST-expressing B16/ST tumor cell line.** B16 mouse melanoma cells were transduced with a lentiviral vector containing a mammalian codon-optimized gene encoding Merkel cell polyomavirus (strain 350) small T antigen (ST) under the control of cytomegalovirus and GFP reporter under EF1 promoter to generate tumorigenic B16/ST tumor cell line. (**A**) Characterization of the transduction of B16/ST tumor cells by flow cytometry analysis after sorting. B16/ST tumor cells (green) or control B16 melanoma cells (purple) were sorted and characterized for GFP expression by flow cytometry analysis. (**B**) Schematic diagram of vaccination schedule for Western Blot analysis. C57BL/6 mice were vaccinated intramuscularly by electroporation three times at 1-week intervals and boosted at the same dose. Western Blot analysis using sera from vaccinated mice was performed 1 month after last vaccination to determine ST protein levels of B16/ST cells. (**C**) ST protein levels determined by Western blot analysis. Membranes were probed with either serum from mice vaccinated with pcDNA3-MCC/ST or anti-β-actin antibody for loading control. *Lane 1,* negative control B16. *Lane 2*, B16-MCC/ST. (**D**) ST RNA levels determined by RT-PCR. *Lane 1,* negative control B16. *Lane 2*, B16-MCC/ST.

B16 mouse melanoma cells were transduced with a lentiviral vector containing a mammalian gene encoding MCPyV ST antigen under the control of cytomegalovirus (CMV) promoter and GFP reporter under EF1 promoter to generate a tumorigenic ST-expressing cell line, B16/ST. Successful transduction of the lentivirus was confirmed by high levels of GFP expression, which allowed us to isolate the transduced tumor cells. As shown in Figure 1A, the ST lentivirus-transduced B16 tumor cells demonstrated significantly higher GFP expression levels compared to non-transduced B16 tumor cells. The GFP-positive cells were further isolated for the characterization of the expression of ST antigen. In order to generate antibodies against ST antigen, mice were vaccinated with pcDNA3-MCC/ST intramuscularly by electroporation according to the schedule shown in Figure 1B. One month following the last vaccination, sera from vaccinated mice were collected for Western blot and RNA analysis. As shown in Figure 1C and D, cell lysates from B16/ST cells demonstrated a specific band consistent with ST antigen protein and RNA levels. In comparison, cell lysates from B16 melanoma cells did not show such a band, indicating the absence of ST antigen. To ensure equal loading of cell lysates, β-actin was used as a control. Thus, our data show the successful creation of a murine B16 tumor cell line that expresses ST, B16/ST.

### Vaccination with MCPyV ST DNA generated an ST-specific CD8+ T cell immune response

**Figure 2:**
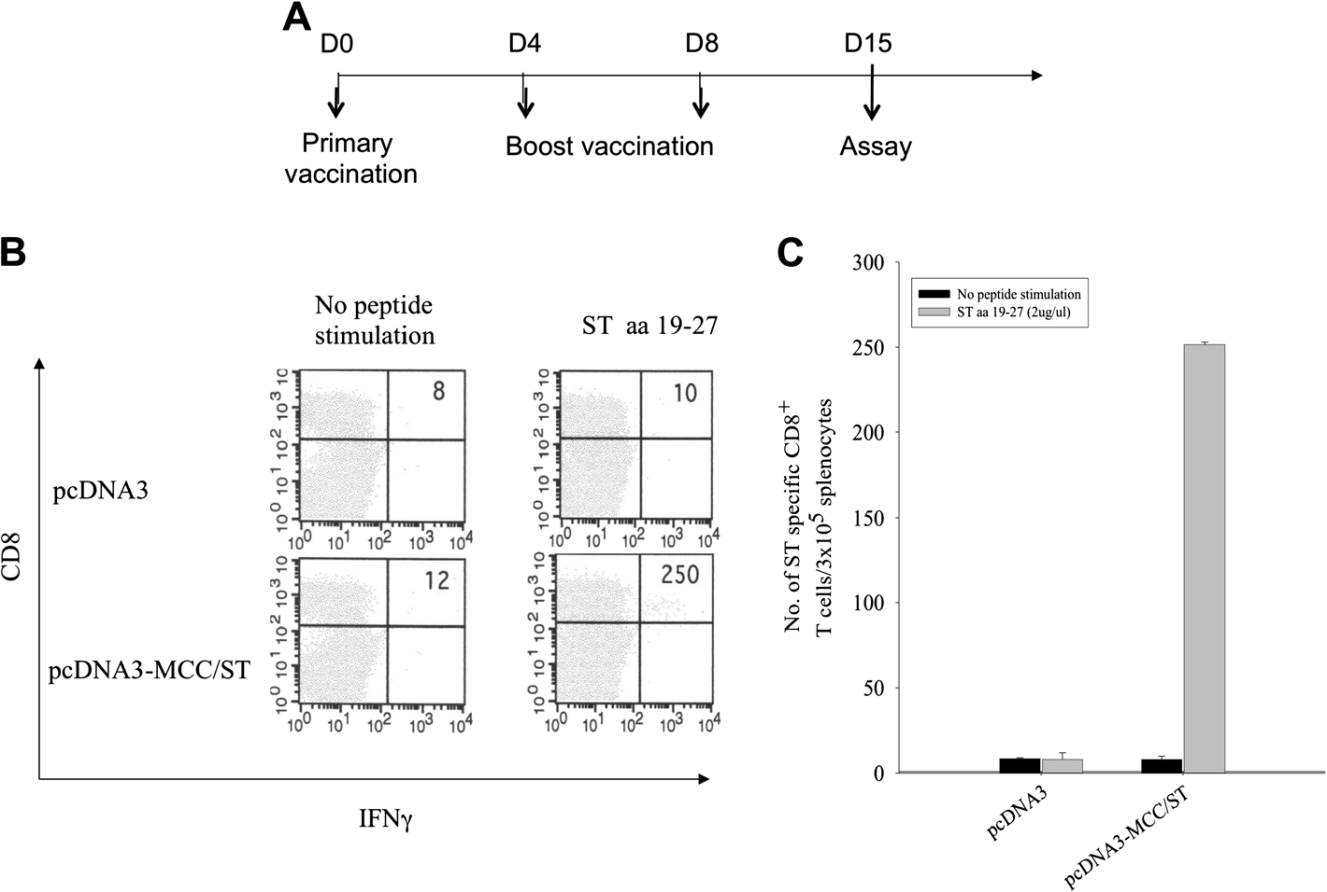
**Characterization of ST-specific CD8+ T cells using splenocytes stimulated with ST peptide aa 19-27.** (**A**) Outline of the vaccination schedule. C57BL/6 mice (5 per group) were immunized with pcDNA3-MCC/ST intradermally with DNA-coated particles using a helium-driven gene gun 3 times at 4-day intervals. Pooled splenocytes from vaccinated mice were collected and cultured *in vitro* with overlapping ST peptide overnight, and stained for intracellular IFN-γ and CD8+ cell surface marker. (**B**) Intracellular cytokine staining followed by flow cytometry analysis to characterize ST-specific CD8+ T cell epitope using a single ST peptide (aa 19-27) from splenocytes harvested from mice vaccinated with pcDNA3-MCC/ST or pcDNA3. (**C**) Bar graph of representative flow cytometry data showing the number of ST-specific CD8+ T cells among 3 × 10^5^ splenocytes. Note that peptide 19-27 activated the highest number of ST-specific CD8+ T cells.

For the characterization of the ST-specific CD8+ T cell immune response, DNA-coated particles were delivered to the shaved abdominal region of each mouse by a helium-driven gene gun. The vaccination schedule is outlined in Figure 2A. The DNA vaccine we generated encoded MCPyV ST aa 1-186 (pcDNA3-MCC/ST). Empty pcDNA3 vector was used as a control. To determine whether vaccination of pcDNA3-MCC/ST or pcDNA3 vaccine was capable of generating ST-specific CD8+ T cell immune responses, intracellular cytokine staining for IFN-γ was performed, followed by flow cytometry. As shown in Figure 2B and C, mice vaccinated with pcDNA3-MCC/ST generated a significant ST-specific CD8+ T cell response when stimulated with the peptide spanning aa 19-27. In comparison, mice vaccinated with the empty vector pcDNA3 did not generate significant numbers of ST-specific CD8+ T cell-mediated responses when stimulated with the same peptides. Thus, our data show that mice vaccinated with MCPyV ST DNA could be generate potent ST-specific CD8+ T cell-mediated immune responses.

### Vaccination with pcDNA3-MCC/ST DNA vaccine provides protection against challenge with ST-expressing tumor cell line, B16/ST

**Figure 3:**
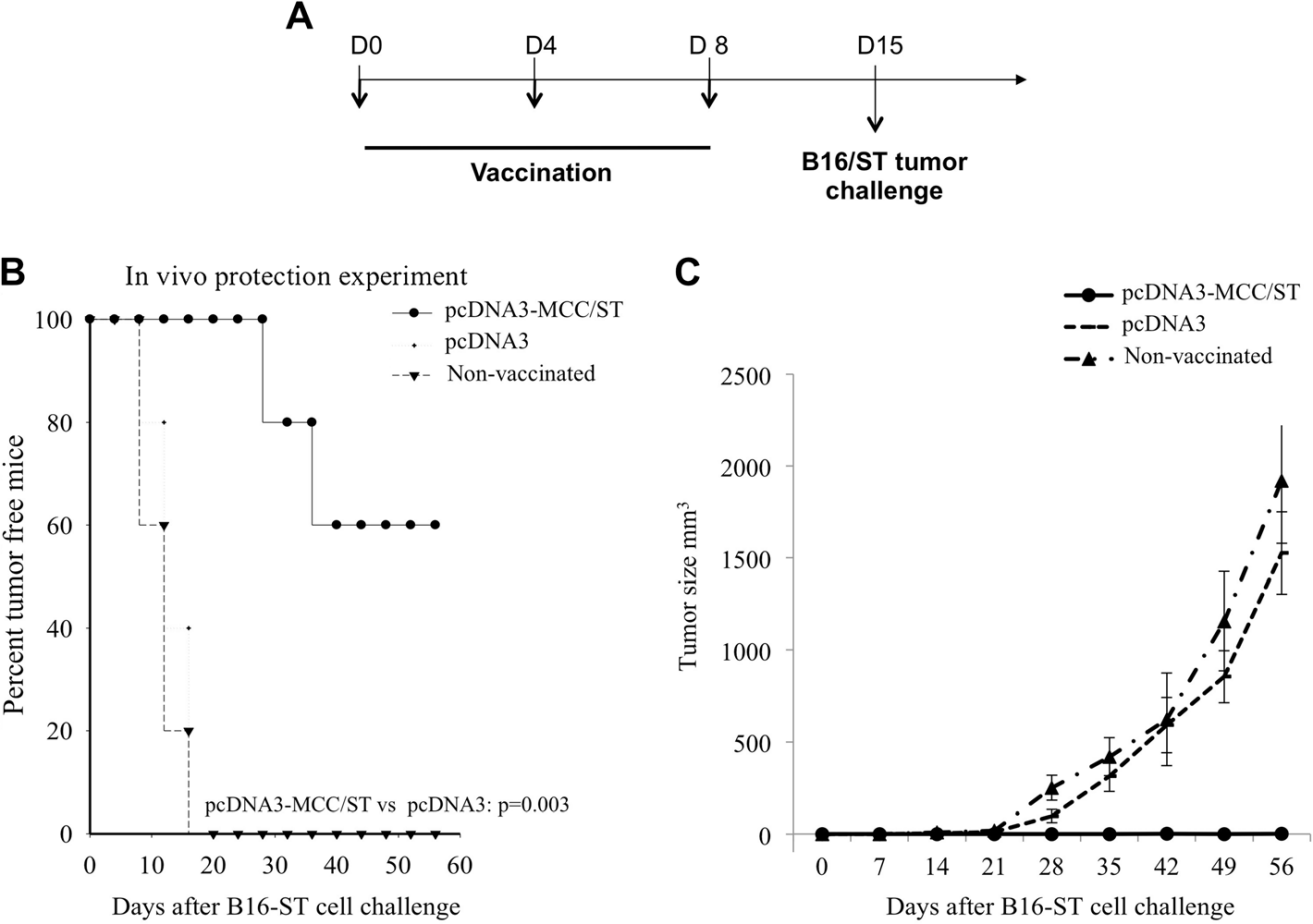
**In vivo tumor protection experiments.** (**A**) An outline of the vaccination schedule using either pcDNA3-MCC/ST DNA vaccine or empty vector control vaccine **pcDNA3**. C57BL/6 mice (5 per group) were immunized with either **pcDNA3**-MCC/ST or **pcDNA3**DNA vaccine intradermally with DNA-coated particles using a helium-driven gene gun 3 times at 4-day intervals. Ten days after the last vaccination, vaccinated mice were challenged subcutaneously in the right flank with B16/ST tumor (1×10^5^ cells/mouse). (**B**) Survival plot depicting the percentage of vaccinated mice surviving following vaccination with either pcDNA3-MCC/ST DNA vaccine or empty vector control pcDNA3. (**C**) Plot depicting tumor volume over time. Tumors were measured with digital calipers and tumor volumes calculated.

Using the vaccination regimen depicted in Figure 3A, we performed *in vivo* tumor protection experiments to analyze the protective antitumor effects of pcDNA3-MCC/ST DNA vaccine. C57BL/6 mice were vaccinated with pcDNA3-MCC/ST DNA or pcDNA3 empty vector as a control. Vaccinated mice were challenged with B16/ST tumor cells subcutaneously one week after the last vaccination. As shown in Figure 3B, mice vaccinated with pcDNA3-MCC/ST had significantly longer survival compared to mice vaccinated with pcDNA3. Furthermore, the tumor volume of mice vaccinated with pcDNA3-MCC/ST was significantly lower than that of mice vaccinated with pcDNA3 (Figure 3C). Thus, our data show that pcDNA3-MCC/ST DNA vaccine generated strong protective antitumor effects in vaccinated mice (pcDNA3-MCC/ST vs. pcDNA3 p=0.002).

### Vaccination with pcDNA3-MCC/ST DNA generates potent therapeutic antitumor effects against ST-expressing tumors

**Figure 4:**
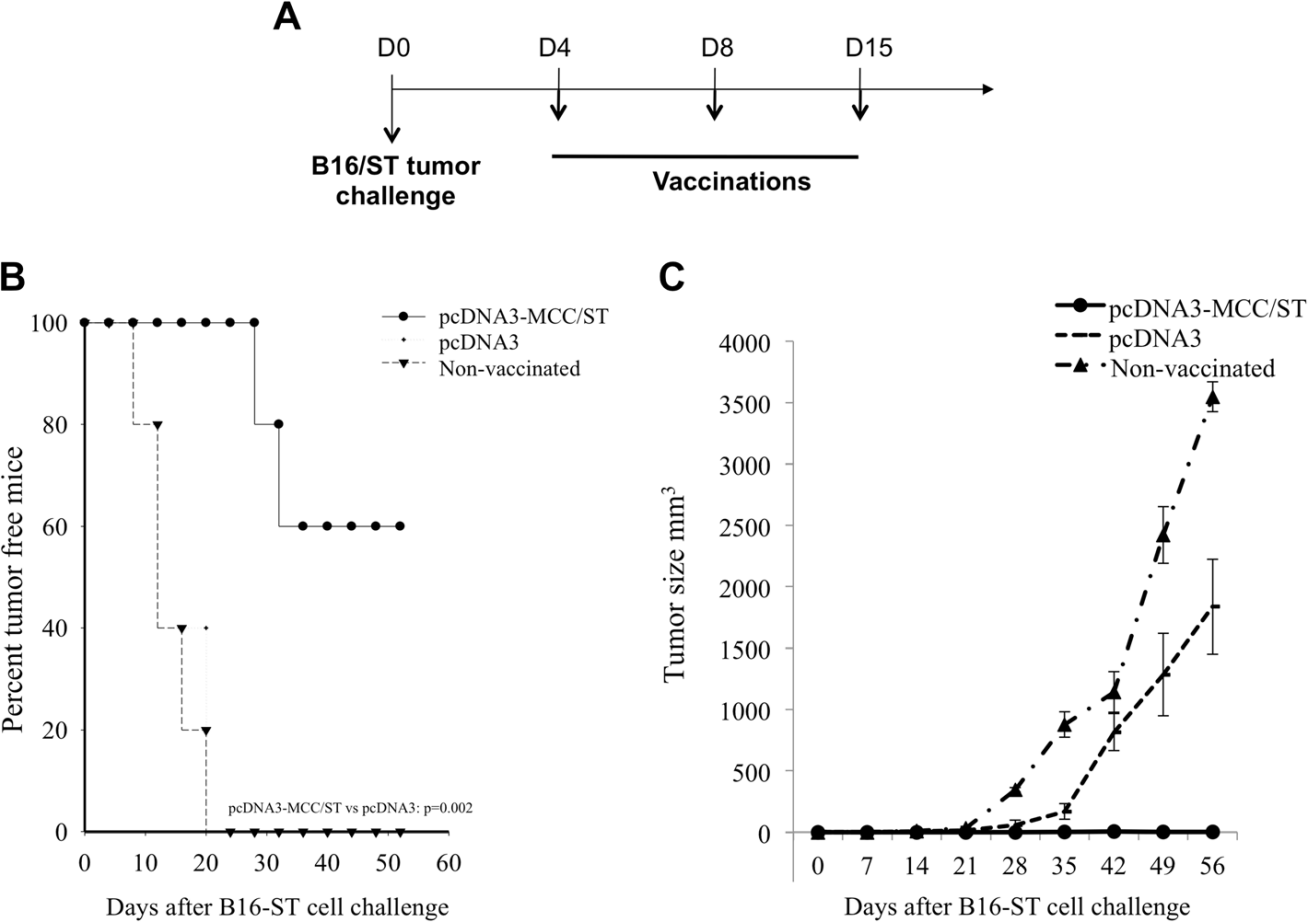
**In vivo tumor treatment experiments.** (**A**) Outline of the treatment regimen with either pcDNA3-ST DNA vaccine or empty vector control pcDNA3. C57BL/6 mice (5 per group) were subcutaneously inoculated with B16/ST tumor (1×10^5^ cells/mouse) in the right flank on D0. Mice were monitored for evidence of tumor growth by visual inspection and palpation. Tumor growth was measured twice a week starting from day 8 after tumor challenge. B16/ST-tumor bearing mice were treated with pcDNA3-MCC/ST or pcDNA3 intradermally with DNA-coated particles using a helium-driven gene gun 3 times at 4-day intervals beginning three days after tumor inoculation. (**B**) Survival plot depicting the percentage of vaccinated mice surviving following vaccination with either pcDNA3-MCC/ST DNA vaccine or control pcDNA3. (**C**) Plot depicting tumor volume over time. Tumors were measured with digital calipers and tumor volumes calculated.

To further determine if the treatment with pcDNA3-MCC/ST DNA vaccine was capable of generating therapeutic antitumor effects, we inoculated C57BL/6 mice first with B16/ST tumor cells and subsequently vaccinated the mice with pcDNA3-MCC/ST or pcDNA3 according to the regimen outlined in Figure 4A. As shown in Figure 4B and C, B16/ST tumor-bearing mice vaccinated with pcDNA3-MCC/ST showed significantly longer survival and lower tumor volume when compared to mice treated with pcDNA3. Therefore, our data indicates that pcDNA3-MCC/ST DNA vaccine can potentially generate therapeutic antitumor effects in B16/ST tumor-bearing mice (pcDNA3-MCC/ST vs. pcDNA3 p=0.002).

### The antitumor effect elicited by pcDNA3-MCC/ST DNA is mediated by CD8+ T cells

**Figure 5:**
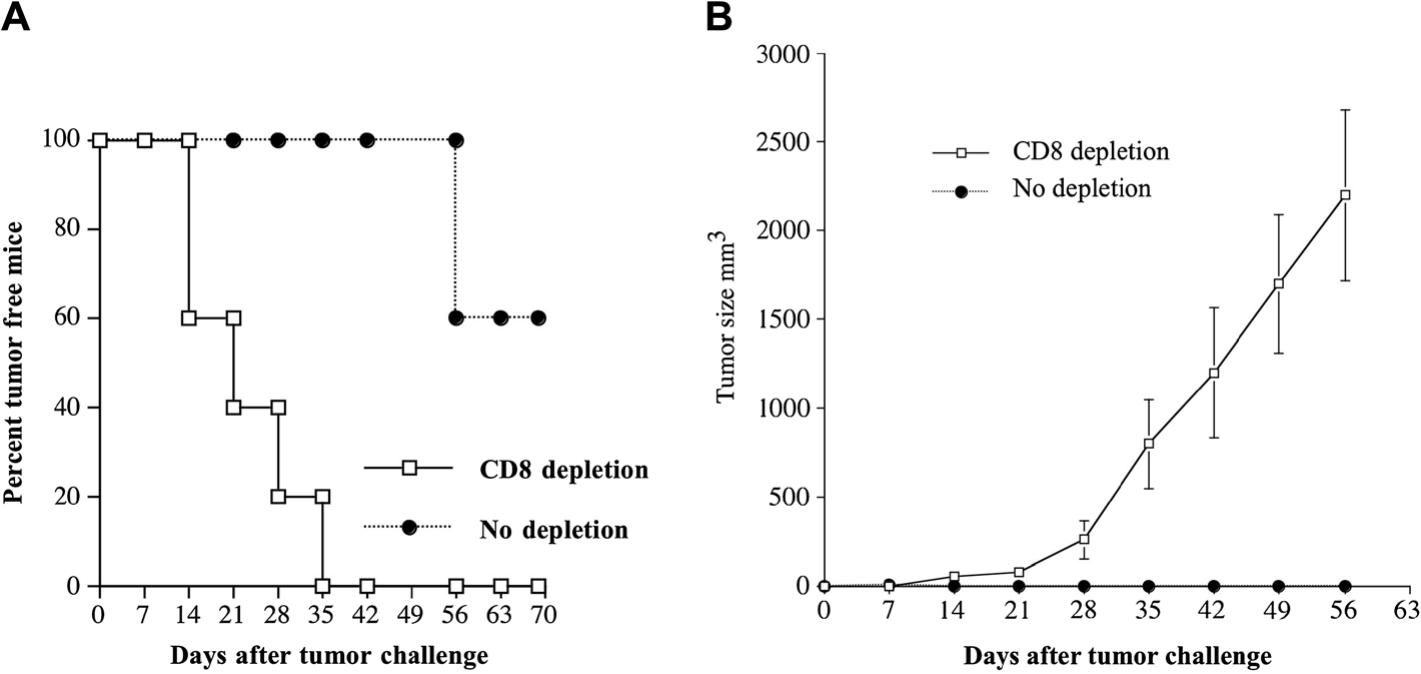
**Characterization of the role of CD8+ T cells in tumor protection elicited by the pcDNA3-MCC/ST vaccine.** Vaccinated mice were boosted two times at the same dose and regimen at one week intervals. Beginning 1 day after last vaccination, vaccinated mice were intraperitoneally injected with anti-CD8 monoclonal antibody other day. Antibody-depleted mice were then challenged with B16/ST tumor (1×10^5^ cells/mouse) subcutaneously in the right flank on day 22 after vaccination. Mice were monitored for evidence of tumor growth by inspection, palpation and tumor size was measured once a week. (**A**) Survival analysis of B16/ST tumor-bearing mice treated with pcDNA3-MCC/ST DNA vaccine. (**B**) Tumor size analysis of B16/ST tumor-bearing mice treated with pcDNA3-MCC/ST DNA vaccine.

In order to examine the impact of CD8+ T cells on the therapeutic antitumor effect elicited by pcDNA3-MCC/ST DNA vaccine against B16/ST tumors, we performed an in vivo antibody depletion assay. C57BL/6 mice were vaccinated with pcDNA3-MCC/ST three times at one week intervals. Beginning on day 15 after the first vaccination, mice were treated with anti-CD8 antibody every other day and then were challenged subcutaneously with B16/ST tumor cells. As shown in Figure 5A, virtually all of the mice depleted of CD8+ T cells developed tumors, compared to only 60% of non-depleted mice. Furthermore, mice treated with anti-CD8 antibody developed significantly larger tumors compared to mice untreated mice. These data suggest that CD8+ T cells are essential for the observed therapeutic antitumor effect against ST-expressing tumors generated by the pcDNA3-MCC/ST DNA vaccine.

## Discussion

In the current study, we successfully created a murine tumor cell line expressing MCPyV small T antigen from the B16 cell line using a CMV promoter, which was confirmed by Western blot. We utilized this ST-expressing tumor cell line to test the efficacy of a DNA vaccine encoding ST (aa 1-186). In B16/ST tumor-bearing mice, this vaccine, pcDNA3-MCC/ST, generated a significant number of ST antigenic peptide (aa 19-27)-specific CD8+ T cells compared to mice vaccinated with empty vector. Furthermore, pcDNA3-MCC/ST markedly enhanced survival in B16/ST tumor-bearing mice in tumor protection and tumor treatment experiments. Furthermore, we have shown that CD8+ T cells play an essential role in the therapeutic effect generated by the pcDNA-MCC/ST vaccine. The formation of an effective vaccine against MCPyV has the potential to advance the field of MCC therapy and may contribute to the control of this severe malignancy through immunotherapy.

The MCPyV ST-expressing tumor cell line established in the current study may be useful the development and testing of other therapeutic strategies for MCPyV-associated malignancies, especially those targeting ST antigen. Considering the oncogenic nature of ST antigen, this tumor cell line could be quite valuable for the characterization of molecular interventions targeting ST antigen.

The current study shows that vaccination with pcDNA3-MCC/ST DNA generates an appreciable ST-specific CD8+ T cell immune response systemically (Figure 2). Previously, we have shown that the level of systemic antigen-specific CD8+ T cells correlates with the level of antigen-specific CD8+ T cells in the tumor loci [9]. Considering that pcDNA3-MCC/ST DNA vaccine elicits potent therapeutic antitumor effects (Figure 4), it is likely that these ST-specific CD8+ T cells are present in tumor loci and contribute to tumor control.

In order to advance the ST-targeting DNA vaccine toward clinical translation, a few adjustments to the immunotherapeutic strategy will have to be made. Firstly, a clinical grade vector will be required to carry the ST DNA in place of pcDNA3. An ideal candidate may be National Gene Vector pNGVL4a, which has been previously used in clinical trials of HPV-16 vaccines [10]. Furthermore, it will be important to determine the most effective method of delivery for the ST DNA vaccine. In the current study, we use both intradermal vaccination by gene gun and electroporation to enhance the efficacy of the DNA vaccine.

While this study provides evidence that a DNA vaccine targeting MCPyV ST antigen can elicit an antitumor immune response in B16 tumor-bearing mice, further experiments will need to be undertaken to improve the understanding of this methodology. It will be important to compare the current treatment with strategies targeting the other antigens of MCPyV, large T antigen and 57 kT antigen. Additionally, therapies targeting multiple MCPyV antigens should also be examined. For example, the ST vaccine used here could be combined with our previously developed large T DNA vaccine for a potentially more potent MCC therapy [11].

In conclusion, this study presents the successful development of a MCPyV ST-expressing tumor cell line as well as an ST-targeting DNA vaccine capable of eliciting antitumor effects. Additionally, further studies are warranted to maximize the efficacy of the ST DNA vaccine as well as promote its advancement toward clinical application. Both of these innovative technologies provide opportunities to develop and test MCPyV-targeted therapies for the control of Merkel cell carcinoma.

## Materials and methods

### Mice

C57BL/6 mice were purchased from National Cancer Institute. All animals were maintained under specific pathogen-free conditions, and all procedures were performed according to approved protocols and in accordance with the recommendations for the proper use and care of experimental animals.

### Constructs

For the generation of the DNA vaccine (pcDNA3-MCC/ST) encoding small T antigen (1-186 aa) of Merkel cell polyomavirus, small T antigen DNA (558 nt) was codon-optimized and synthesized from GeneScript Corporation (Piscataway, NJ) and cloned into NheI and NotI sites of pcDNA3 (Invitrogen, Carlsbad, CA). For generation of lentivirus encoding encoding truncated small T antigen, the small T antigen DNA was cloned into NheI/NotI sites of pCDH1-EF1-GFP vector (System Bioscience, Mountain View, CA) to generate pCDH1-STop-EF1-GFP. The vector contains two promoters, CMV promoter for small T antigen and EF1 promoter for GFP expression.

### Peptides

Previously, using 15 over-lapping amino acids spanning aa 1-258 of the large T (LT) antigen of MCPyV, we identified the large T immunogenic epitope occurring at aa 19-27 (IAPNCYGNI) [11]. The small T antigen has a peptide region that overlaps with that of the LT antigen, which includes the epitope, MCPyV ST aa 19-27 (IAPNCYGNI), as a region generating an ST-specific CD8+ T cell immune response.

### Cell lines

Small T and GFP-expressing B16F10 (B16/ST) were generated by transduction with a lentiviral vector containing small T antigen and GFP. Lentiviral vector pCDH1-STop-EF1-GFP, pCMVΔR8.91, and pMDG were transfected into 293T cell line using lipofectamine (Invitrogen) and the virion-containing supernatant was collected 48 h after transfection. The supernatant was then filtered through a 0.45 mm cellulose acetate syringe filter (Nalgene, Rochester, NY) and used to infect B16F10 cells in the presence of 8 μg/ml Polybrene Sigma-Aldrich, St Louis, MO). Transduced cells were isolated using preparative flow cytometry with GFP signal.

### Western blot analysis

Whole cell protein lysates were obtained from both B16 and sorted B16-MCC/ST cells and were extracted using M-PER mammalian protein extraction reagent (Thermo Scientific, Rockford, IL) containing complete protease inhibitor tablet (Roche Diagnostic, Indianapolis, IN). Equal amounts of proteins (30 μg) were loaded and separated by 4-15% gradient ready SDS-PAGE gel. The gels were transferred to a polyvinylidene difluoride membrane (Bio-Rad, Hercules, CA). Blots were blocked with phosphate-buffered saline-Tween20 (PBST) containing 5% non-fat milk for 15 minutes at room temperature. Membranes were probed with anti-β actin (Sigma, St. Louis, MO) at 1:5,000 dilution or sera from mice vaccinated pcDNA3-MCC/ST at 1:100 dilution in PBST for 2.5 hours, washed several times with PBST, and then incubated with rabbit anti-mouse IgG conjugated to HRP (Invitrogen) at 1:5,000 dilution in PBST containing 2.5% nonfat milk. Membranes were washed several times with PBST and developed using enhanced Hyperfilm-enhanced chemiluminescence (Amersham, Piscataway, NJ).

### RT-PCR

RNA was extracted by TRIZOL (Invitrogen) from B16 and B16/ST cell lines. PCR was performed using the Superscript One-Step RT-PCR Kit (Invitrogen) using 1 μg of the total RNA. Sequences of primers for small T antigen and actin were as follows: small T-F (5′-CAAGGTTCTGCAGGGGACCAGGATG -3′), small T-R (5′ GAACAGGTGCAGGTGCAGCAGGCAG -3′), actin-F (5′-ACTGGGACGACATGGAGAAG -3′), and actin-R (5′-GGGGTGTTGAAGGTCTCAAA -3′). The reaction conditions for small T antigen was 1 cycle (94°C, 30 sec), 35 cycles (94°C, 30 sec; 55°C, 30 sec; 72°C, 30 sec), and 1 cycle (72°C, 10 min). The reaction conditions for actin was similar except that amplification was repeated for 25 cycles. The products were analyzed by electrophoresis on a 1.5% agarose gel containing ethidium bromide.

### DNA vaccination

In order to obtain sera for Western blot analysis, DNA vaccine application was mediated by electroporation. The Electroporation Delivery System (BTX, San Diego, CA) was used according to the manufacturers protocol and as described previously [12]. The vaccine was administered three times at 1-week intervals and boosted at the same dose using 40 μg of DNA per dose. C57BL/6 mice (5 per group) were vaccinated with pcDNA3-MCC/ST.

For the remaining experiments, vaccine application was performed using DNA-coated particles, which were administered intradermally using a helium-driven gene gun (Bio-Rad Laboratories, Hercules, California) using methods previously described [13]. Briefly, C57BL/6 mice (5 per group) were vaccinated with pcDNA3 or pcDNA3-MCC/ST DNA-coated particles delivered to the shaved abdominal region of each mouse using a helium-driven gene gun with a discharge pressure of 400 psi. Mice were initially immunized at a dose of 2 μg of each DNA vaccine and boosted with the same dose two times at 4-day intervals.

### In vivo tumor protection experiments

C57BL/6 mice (5 per group) were immunized with either pcDNA3-MCC/ST or pcDNA3 empty vector DNA vaccine by gene gun as described above. One week after the last vaccination, vaccinated mice were challenged subcutaneously in the right flank with B16/ST tumor cells (1×10^5^ cells/mouse). Mice were monitored twice per week for survival.

### In vivo tumor treatment experiments

C57BL/6 mice (5 per group) were subcutaneously inoculated with B16/ST tumor (1×10^5^ cells/mouse) in the right flank on D0. B16/ST-tumor bearing mice were treated with pcDNA3-MCC/ST or pcDNA3 intradermally by a helium-driven gene gun three times at 4-day intervals beginning three days after tumor inoculation. Mice were monitored twice per week for survival.

### In vivo antibody depletion experiment

C57BL/6 mice (5 per group) were vaccinated by the gene gun method with pcDNA3-MCC/ST vaccine on day 0. Vaccinated mice were boosted two times at the same dose and regimen at 1 week intervals. One day after the last vaccination, mice were intraperitoneally injected with anti-CD8 antibody every other day. Antibody-depleted mice were then challenged with B16/ST tumor cells (1×10^5^ cells/mouse) subcutaneously in the right flank on day 22. Mice were monitored for evidence of tumor growth by inspection and palpation. Tumor size was measured twice a week.

### Flow cytometry

Detection of cellular surface CD8a and intracellular IFN-γ was performed using flow cytometry as described previously [14]. Briefly, the cells were incubated overnight with 1 μg/ml of GolgiPlug (BD Pharmingen) in the presence of 2 μg/ml of small T antigen overlapping peptides. After washing with FACScan buffer, the cells were stained with phycoerythrin-conjugated anti-mouse CD8a antibody. The cells were then incubated with BD cytofix/cytoperm solution (BD Pharmingen) followed by staining with FITC-conjugated anti-mouse IFN-γ antibody. Flow cytometry analysis was performed on a Becton-Dickinson FACSCalibur with CELLQuest software (BD Biosciences, Mountain View, CA).

### Statistical analysis

Comparisons between individual data points were made using a Student’s *t*-test. Kaplan-Meier survival curves were applied for protection and tumor treatment experiments; for differences between curves, *p-*values were calculated using the log-rank test. The values of *p*<0.05 were considered significant.

## Competing interests

The authors declare that they have no competing interests.

## Authors’ contributions

TW, RV and CH conceived and designed experiments. BG, LH, and YT performed experiments. BG analyzed the data. BG, TW, RV and CH prepared the manuscript. All authors read and approved the final manuscript.
